# Urban Transformations and Health: Methods for TrUST—a Natural Experiment Evaluating the Impacts of a Mass Transit Cable Car in Bogotá, Colombia

**DOI:** 10.3389/fpubh.2020.00064

**Published:** 2020-03-10

**Authors:** Olga L. Sarmiento, Diana Higuera-Mendieta, Maria A. Wilches-Mogollon, Luis A. Guzman, Daniel A. Rodríguez, Ricardo Morales, Daniela Méndez, Claudia Bedoya, Mario Linares-Vásquez, Maria Isabel Arévalo, Eliana Martínez-Herrera, Felipe Montes, Jose D. Meisel, Andrés F. Useche, Elizabeth García, Camilo A. Triana, Andrés L. Medaglia, Philipp Hessel, Julian Arellana, Carlos Moncada, Abby C. King, Ana V. Diez Roux

**Affiliations:** ^1^School of Medicine, Universidad de Los Andes, Bogotá, Colombia; ^2^Department of Industrial Engineering, School of Engineering, Universidad de Los Andes, Bogotá, Colombia; ^3^Department of Civil and Environmental Engineering, School of Engineering, Universidad de Los Andes, Bogotá, Colombia; ^4^College of Environmental Design and Institute for Transport Studies, University of California, Berkeley, Berkeley, CA, United States; ^5^Systems Engineering and Computing Department, School of Engineering, Universidad de Los Andes, Bogotá, Colombia; ^6^National School of Public Health, Research Group of Epidemiology, Universidad de Antioquia, Medellín, Colombia; ^7^Facultad de Ingeniería, Universidad de Ibagué, Ibagué, Colombia; ^8^Fundación Santa Fe de Bogotá, Bogotá, Colombia; ^9^School of Government, Universidad de Los Andes, Bogotá, Colombia; ^10^Department of Civil and Environmental Engineering, Universidad del Norte, Barranquilla, Colombia; ^11^Facultad de Ingeniería, Universidad Nacional de Colombia, Bogotá, Colombia; ^12^Department of Epidemiology & Population Health, Stanford University School of Medicine, Stanford, CA, United States; ^13^Stanford Prevention Research Center, Department of Medicine, Stanford University School of Medicine, Stanford, CA, United States; ^14^Urban Health Collaborative, Dornsife School of Public Health, Drexel University, Philadelphia, PA, United States; ^15^Department of Epidemiology and Biostatistics, Dornsife School of Public Health, Drexel University, Philadelphia, PA, United States

**Keywords:** cable car, impact evaluation, Latin America, urban health, transport, physical activity, Citizen's Science

## Abstract

**Background:** Cable cars provide urban mobility benefits for vulnerable populations. However, no evaluation has assessed cable cars' impact from a health perspective. TransMiCable in Bogotá, Colombia, provides a unique opportunity to (1) assess the effects of its implementation on the environmental and social determinants of health (microenvironment pollution, transport accessibility, physical environment, employment, social capital, and leisure time), physical activity, and health outcomes (health-related quality of life, respiratory diseases, and homicides); and (2) use citizen science methods to identify, prioritize, and communicate the most salient negative and positive features impacting health and quality of life in TransMiCable's area, as well as facilitate a consensus and advocacy-building change process among community members, policymakers, and academic researchers.

**Methods:** TrUST (In Spanish: Transformaciones Urbanas y Salud: el caso de TransMiCable en Bogotá) is a quasi-experimental study using a mixed-methods approach. The intervention group includes adults from Ciudad Bolívar, the area of influence of TransMiCable. The control group includes adults from San Cristóbal, an area of future expansion for TransMiCable. A conceptual framework was developed through group-model building. Outcomes related to environmental and social determinants of health as well as health outcomes are assessed using questionnaires (health outcomes, physical activity, and perceptions), secondary data (crime and respiratory outcomes) use of portable devices (air pollution exposure and accelerometry), mobility tracking apps (for transport trajectories), and direct observation (parks). The Stanford Healthy Neighborhood Discovery Tool is being used to capture residents' perceptions of their physical and social environments as part of the citizen science component of the investigation.

**Discussion:** TrUST is innovative in its use of a mixed-methods, and interdisciplinary research approach, and in its systematic engagement of citizens and policymakers throughout the design and evaluation process. This study will help to understand better how to maximize health benefits and minimize unintended negative consequences of TransMiCable.

## Introduction

The way cities are designed affects the health of their populations (1). Specifically, urban and transport planning can have direct and indirect effects on non-communicable diseases, mental health, exposure to air pollution, physical activity behaviors, and well-being (1). Therefore, the health sector should advocate for integrated multisector city planning that prioritizes health-related outcomes, particularly in rapidly urbanized low-to-middle-income countries (2).

Latin America is one of the most violent and urbanized regions in the world, with large populations living in informal settlements and substantial social and spatial inequalities (3, 4). Together, these social disadvantages manifest themselves in health inequities within and across cities (5). Latin America is also a major hub for innovation in urban transport and mobility policies (6). However, the health impacts of these policies rarely have been evaluated.

Cable cars for urban mobility are an emerging innovation from Latin America. They were initially popular in tourist destinations such as ski resorts and mountain tops. More recently, they are being used as a mass transport alternative for day-to-day mobility of local residents (7). Since 1959, at least 24 cities worldwide (Latin America, North America, Asia, Africa, and Europe) have implemented cable cars as part of their transit systems (8–10). Since 2004, the fastest expansion has occurred in Latin American cities. In Colombia, Medellín's cable car was a pioneer because it deliberately integrated the service to the city's existing mass transit system (11). The newest cable car system in Latin America is TransMiCable in Bogotá, Colombia. Following Medellín's example, TransMiCable is linked to a socio-economic inclusive urban development strategy implemented in the area in which it operates.

Despite the increasing popularity of cable cars, only the cable cars in Medellín (Colombia) and La Paz-El Alto (Bolivia) have been evaluated (11–18). Findings from these evaluations have shown relevant transport and environmental effects such as reductions in travel time (13, 14) and increased energy efficiency (15), and improved access to social services (14). However, the effect cable cars on health and social determinants of health has been limited and included increased social cohesion (11, 12), decreased homicide rates (12, 16), reduction of unemployment (14), higher resident perceptions of inclusion (17, 18), and higher resident self-esteem (17).

However, these systems have not been evaluated concerning impacts on physical activity or air pollution. The complementary interventions on recreational areas and parks offer opportunities for further synergies that impact both physical activity and air pollution. Current evidence shows that public transit interventions can impact physical activity levels (19). Furthermore, the creation of new bus rapid transit (BRT) routes, as well as BRT fleet age and transport proximity, have been associated with changes in air pollution or respiratory health (20). Moreover, the implementation of parks, parks renovations and parks programs have been associated with increases in physical activity during leisure time and with improvements in quality of life (21).

### The Historical Context of TransMiCable

Bogotá, the capital of Colombia, has 7.2 million residents (22). During the late 1940s to mid-1950s, amid the industrial development of Bogotá and the upsurge in armed conflict in the country, massive rural-to-urban migration led to informal subdivisions of land (23, 24). Rapid urbanization and high land prices drove marginalized populations to urban peripheral areas. Affordable land was not only distant from activity centers but also characterized by steep hillsides prone to landslides, where land development is often informal (25). As a result, the population living in these peripheries in Bogotá are deprived of public services, have low access to job opportunities, limited urban amenities, and constrained transport options (26, 27).

Ciudad Bolívar is an administrative area of Bogotá, with 616,300 inhabitants in 252 neighborhoods, exemplifying the struggles of populations living in self-built settlements in the peripheries of similar Latin American cities. Ciudad Bolívar has been home to victims of the forced internal displacement generated by different waves of violence in the country (28). It was not until 1984 that Ciudad Bolívar was annexed and considered within the limits of Bogotá. In fact, multiple confrontations led by the Ciudad Bolívar community have helped in the creation of common rules of occupancy that have been among the core mechanisms to achieve recognition from the local government and the provision of public services (29–31).

Despite these advances, El Paraíso and El Mirador del Paraíso, two of the most distant neighborhoods in Ciudad Bolívar, have faced constant struggles for public services, including transport access (32). Unplanned urbanization, migration, illegal subdivisions of land, land disputes, violence, and poor health outcomes characterize this area (25). Despite these challenges, these neighborhoods have overcome many of the impediments posed by the conflict to work collectively for a new and more promising present and future (28). In 2007, the community leaders of these neighborhoods started a social mobilization and advocacy effort for a cable car inspired by the case of Medellín (33). This collective action was vital for the approval of the local budget and for continuing its construction despite government change.

In this context, the implementation of TransMiCable offers a unique opportunity to assess the effects of the cable car project on the environmental and social determinants of health, physical activity, and health outcomes. The cable car is embedded in a socio-economic inclusive urban development program aimed at reducing socioeconomic inequalities. Understanding the health impacts of the implementation of TransMiCable will inform global and country-specific efforts toward sustainable transport, particularly for emerging and increasingly attractive options such as cable cars in marginalized areas. Furthermore, this knowledge is also important for several development pacts and covenants such as New Urban Agenda (34), the Sustainable Development Goals (35), and some additional global, country, and city-specific plans and initiatives (36–41).

This paper aims to describe the study design and methods of the project called Urban Transformations and Health: The case of TransMiCable in Bogotá (or TrUST– in Spanish: Transformaciones Urbanas y Salud: el caso de TransMiCable en Bogotá). An initiative supported by the SALURBAL (Salud Urbana en América Latina, or Urban Health in Latin America) Project (42), TrUST is being undertaken by an interdisciplinary group of researchers drawing on expertise from public health, psychology, transport, geography, urban design and planning, anthropology, complex systems, industrial engineering.

## Methods and Analysis

### Aims

The aims of the study are the following:

(1) To assess the effects of TransMiCable's implementation on the environmental and social determinants of health (micro-environment pollution, physical environment perceptions, access to recreational and cultural facilities, transport accessibility, employment, social capital, and leisure time), physical activity (leisure and transport physical activity), and health outcomes (health-related quality of life, respiratory diseases, and homicides) in the area of influence of TransMiCable in Ciudad Bolívar by comparing the intervention area to San Cristóbal, a similar area designated for future expansion for TransMiCable.
(2) To use citizen science methods to identify, prioritize, and communicate the most salient negative and positive features impacting health and quality of life in TransMiCable's area, as well as facilitate a consensus and advocacy-building change process among community members, policymakers, and academic researchers.

### The Intervention

TransMiCable was inaugurated in December 2018 and comprises a single 3.43 km line with four stations connecting it to the city's BRT system (TransMilenio). Before the cable car, users took different forms of motor-vehicle based public transport (either publically provided or by semi-formal, private operators) to either connect to or reach different parts of the city. Due to circuitous streets and steep hills, travel times to TransMilenio were high, averaging 62 min from the furthest neighborhoods and 32 min from the nearest cable station. For those users connecting to TransMilenio, the cable car brings expected travel time reductions of at most 80%.

**Figure 1:**
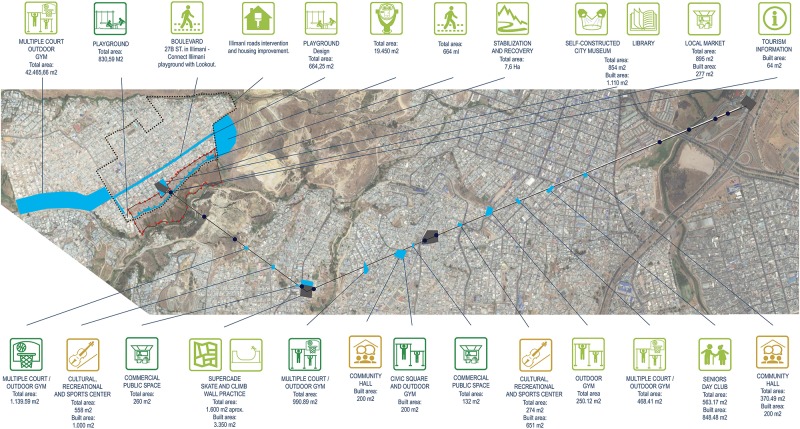
Location of programs and infrastructure projects of TransMiCable. Source: Infographic from the city of Bogotá Planning Department. Reprint with permission from Martín Anzellini García-Reyes, an officer of the Bogotá Planning Department.

The construction of the cable car is accompanied by a broader urban development program for the area that includes the following (see Figure 1): (1) facilities for recreation and cultural activities (two trails; 11 parks; one history museum; one library and two culture, sports and recreation centers); (2) three community centers; (3) three local market facilities; (4) a tourism office; (5) a citizen service office; (6) a program to support physical improvements to homes; and (7) a project to reduce geomorphological hazards. The management and activities developed within these programs follow a participatory process that includes community members and an intersectoral group of city employees. The total cost of TransMiCable and its comprehensive urban development program is around 109 million USD.

### Co-creation of a Conceptual Framework

**Figure 2:**
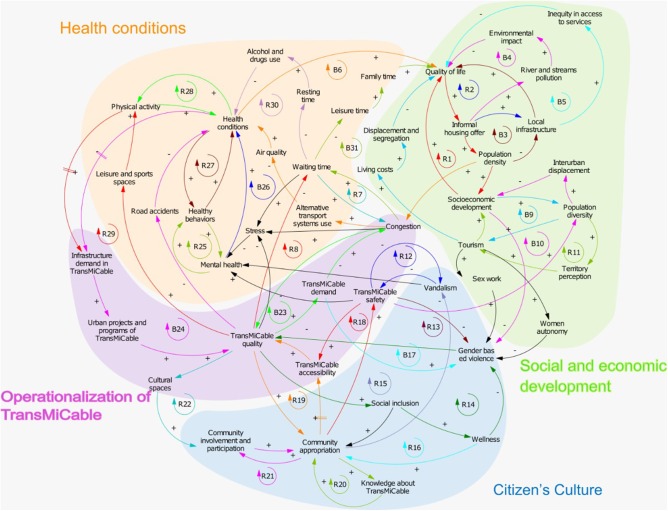
Causal Loop Diagram Depicting the Conceptual Framework of TrUST.

To develop the study framework, we followed two steps. First, we identified a theory-driven conceptual framework developed by Giles-Corti that illustrates linearly direct and indirect pathways through which urban and transport planning influence health and well-being (1). These linear pathways were adapted to show the specific potential impacts of the implementation of TransMiCable and its concurrent urban development projects. Specifically, our framework includes as *urban system policies* the implementation of the transport system of TransMiCable and the 16 projects of the urban development program. Second, this conceptual framework was complemented through a group-model building (GMB) workshop using systems dynamics (43) (Figure 2).

The workshop included 31 stakeholders of nine organizations. Participants belonged to the following sectors: Academia (Universidad de Los Andes and Universidad de Ibagué); government (the National Institute of Health; Bogotá's Secretariats of Health, Habitat, Women, and Social Integration; the Institute of Sports and Recreation of Bogotá; TransMilenio S.A, (the managing and coordinating entity of the public transport system of Bogotá, including TransMiCable); and civil society (two community leaders from Ciudad Bolívar, who attended after an open invitation to community leaders). After the workshop, we contacted each participant to (1) shed light on feedback loops depicted in the causal loop diagram, and (2) clarify terminology in order to be accurate with the jargon of each discipline and sector. Eleven of the participants from three sectors reviewed the diagram.

The causal loop diagram shows the dynamics of 32 feedback loops (19 of reinforcement and 12 of balance) grouped into four domains relevant to understanding the plausible impact of TransMicable (Figure 2, Appendix 1). The first domain corresponds to health conditions in which social dynamics favor physical activity promotion and the dynamics of transport that could influence mental health and leisure activities. The second domain corresponds to social and economic development in which social dynamics could have an impact on mobility, inter-urban displacement, and quality of life. The third domain corresponds to citizen's culture, involving community participation and ownership, and reinforcement of inclusive behaviors that nurture well-being and reduce vandalism and gender-based violence. The fourth domain corresponds to the operationalization of TransMiCable, which entails the provision of an efficient service.

### Study Design

**Figure 3:**
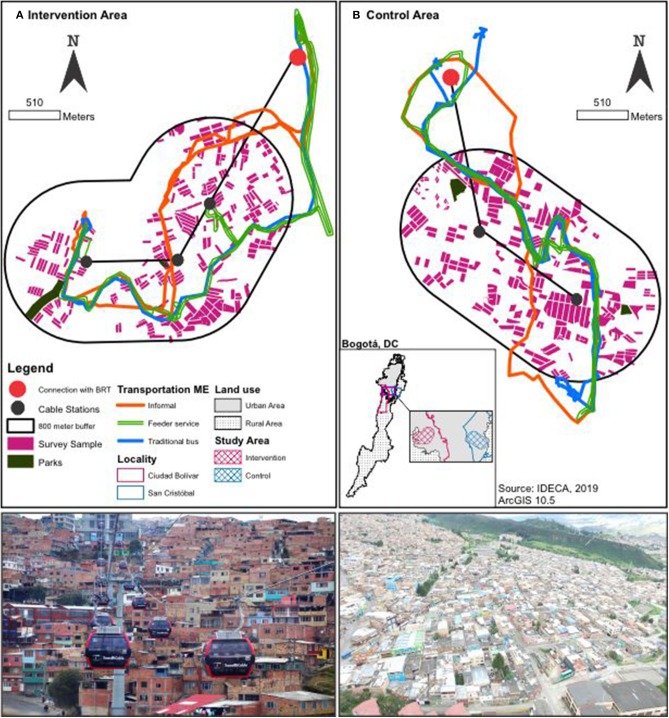
Intervention area in Ciudad Bolívar (**A**, left) and control group in San Cristóbal (**B**, right).

This is a prospective, quasi-experimental study using a mixed-methods approach. In all analyses, we are contrasting the “intervention area” with a “control area.” The intervention area comprises a set of households located in blocks and neighborhoods within an 800-m airline buffer around each of the current TransMiCable stations (Figure 3A). This distance exceeds traditional walking standards used in transport studies because we hypothesized that low-income residents would walk long distances to access this time and cost-saving transport intervention. The control area includes households in the future area of influence of TransMiCable in San Cristóbal (Figure 3B). The potential area of influence of TransMiCable in the control group comprises a set of households located in blocks and neighborhoods within an 800-m buffer around potential stations of the future TransMiCable line.

The control area is one of the nine projected cable cars for the city of Bogotá. San Cristóbal is an adequate control group for the following reasons: (1) among a list of nine projected cable cars, San Cristóbal's cable was the first prioritized by the local administration, after Ciudad Bolívar's cable; (2) there are geographical barriers separating intervention and control groups, limiting contamination; (3) both groups have similar topography characteristics and similar cable car system characteristics (length, elevation difference); and (4) San Cristóbal and Ciudad Bolivar are mostly from low socio-economic status and has the second-highest crime indicators in Bogotá after Ciudad Bolívar.

A variety of hypotheses regarding the impact of the intervention will be tested in intervention and control areas before and after the intervention. The measurements, unit of analysis of indicators, and hypotheses are shown in Table 1. Hypotheses involving impacts on individuals will be tested in analyses collecting data at the individual level on a sample of residents. Hypotheses regarding impact on air pollution exposures and inhaled doses will be tested by contrasting individual-level exposures in selected transport-related micro-environments that resemble the main modes of transportation of the population in intervention and control areas. Hypotheses regarding crime and respiratory illness, as well as changes to built environments, will be tested by comparing rates or built environment features in the intervention and control areas. Hypotheses related to transport trajectories will be tested in a convenience sample of the intervention and control group. Hypotheses related stated transport preferences will be tested in a random sample of the intervention group. Lastly, hypotheses regarding the impact on parks will be tested by comparing two parks in the intervention area with two parks in the control area.

**Table 1:** Measurements, unit of analysis of indicators, and hypotheses of the TrUST study.

**Measurement**	**Unit of analysis**	**Measurement instrument**	**Impact measurement**	**Hypotheses**
**Environmental determinants of health**				
Exposure and inhaled doses of fine particulate matter (PM_2.5_), equivalent black carbon (eBC), and carbon monoxide (CO)	Transport microenvironments: (1) roadside passenger waiting area of BRT station, (2) main feeder line to the BRT system, (3) regular bus serving each area, 4) a semi-formal public transport vehicle, and 5)TransMicable cabins	DustTrak models 8520 and 8530, TSI Inc. MN, USA, Personal Environmental Monitors (PEM), (SKC Inc. PA, USA), Aethalometers (AE51, MicroAeth, CA, USA) and (DeltaOhm, P37AB1347 SICRAM probe)	Change of mean concentration to pollutants Change of inhaled dose of pollutants.	Ha: The estimated concentration and inhaled dose of pollutants decrease in the population that changed modal share from motorized vehicles to TransMiCable.
Neighborhood perceptions	Individuals in households	TrUST Survey	Change in the mean score of neighborhood perceptions (safety, aesthetics, and satisfaction with the transport system)	Ha: Neighborhood perceptions of safety, and aesthetics and satisfaction with transport improve in the area of influence of TransMiCable when compared to the observed area of San Cristóbal.
The proportion of the area that includes recreational and cultural facilities, community centers, and local markets.	800-m buffer around each of the current and projected TransMiCable in intervention and control areas.	IDECA spatial data of infrastructure	Change in the proportion of area for recreational and cultural facilities, community centers, and local markets	Ha: The proportion of area for recreational and cultural activities increases in the area of TransMiCable compared to the proportion in San Cristóbal.
Park quality, occupation and activity level	Two parks in the intervention area and two parks in the control area.	PARA and SOPARC	Change in the quality score of parks (PARA) % change in parks occupation % change in parks activity level	Ha: The mean PARA score increases in parks in the area of TransMiCable compared to the mean score in San Cristóbal.Ha: The occupation and observed levels of physical activity in the parks of the area of TransMiCable increase compared to the occupation and levels of San Cristóbal.
Travel time, costs, demand, and modal share	Individuals in households	TrUST Survey	Change in mean travel time Change in mean travel cost	Ha: Travel time and cost for trips to work/study destinations decreases when modal share changes from motorized vehicles to TransMiCable.
Transport trajectories and activity places	Individuals in households who accepted using MOVES app and/or Muévelo app	MOVES and Muévelo app	Change in the mean number of activity places	Ha: The number of visits to activity places increases in the new users of TransMiCable compared to the number of visits in San Cristóbal.
Origin-destination matrix over time in Bogotá	Bogotá, Ciudad Bolívar and San Cristóbal areas.	Fare card data from TransMilenio	Change in OD matrix values	Ha: The percentage of trips to and from the main portal (Tunal) in the area of TransMicable increases after the implementation of the cable. The percentage of trips to and from the main portal (20 de Julio) in the area of San Cristóbal will not change.
Transit transport equity	Bogotá metropolitan localities.	Transit Opportunity Index	Change in the TOI values	Ha: The proportion of the TOI (measure of transport access) from and to Ciudad Bolívar after the implementation of TransMicable will increase relative to the TOI of the other localities in the metropolitan area.
**Social determinants of health**				
Employment and leisure time	Individuals in households	TrUST Survey	Change in the unemployment rate	Ha: The unemployment rate decreases in the area of influence of TransMiCable in comparison to the rate of the observed area of San Cristóbal.
Social capital	Individuals in households	TrUST Survey	Change in social capital classes (dimensions: social networks, trust, cooperation, empowerment)	Ha: The classes of social capital with the highest social networks, trust, cooperation, and empowerment dimensions increase in the area of influence of TransMiCable compared to the classes of San Cristóbal.
**Physical activity**				
Physical activity for transport and leisure time	Individuals in households	TrUST Survey (International Physical Activity Questionnaire)	Change in minutes of physical activity for transport Change in minutes of physical activity for leisure time	Ha: The mean minutes of leisure-related physical activity increases in the new users of TransMiCable compared to the mean minutes of non-users of TransMicable and the residents of San Cristóbal. Ha: The mean minutes of walking for transport increase in the new users of TransMiCable compared to the mean minutes of non-users of TransMicable and the residents of San Cristóbal.
Objective physical activity	Individuals in households for whom we could collect data until December 2018	ActiGraph GT3X or GT3X+	Mean minutes change of physical activity	Ha: The mean minutes of moderate-to-vigorous physical activity increase in the new users of TransMiCable compared to the mean minutes of non-users of TransMicable and the residents of San Cristóbal.
**Health outcomes**				
Health-related quality of life	Individuals in households	TrUST Survey (The WHOQOL-brief instrument)	Mean score change in QOL scores in environmental, physical, social, and psychological domains	Ha: The mean score in QOL increases in the population living in the area of influence of TransMiCable compared to the scores of residents in San Cristóbal.
Respiratory diseases	Individuals in households	TrUST Survey and secondary database of SIVIGILA	Change in the number of reported respiratory diseases in <5 years old and >60 years old	Ha: The number of respiratory diseases reported in the population living in the area of influence of TransMiCable decreases compared to the number in residents of San Cristóbal.
Crime	Individuals in households	Secondary data from the homicides from the National Police Records	Change in the homicide rate	Ha: The number of homicide rate decreases in the area of influence of TransMiCable compared to the rate in San Cristóbal.
**Qualitative component**				
The historical context of the intervention	Community leaders	Semi-structured interviews		
Citizen science “by the people” Our voice model	A convenience sample of individuals living in the more distant neighborhoods in Ciudad Bolívar and San Cristóbal	The Stanford Healthy Neighborhood Discovery Tool, and meetings with the community and stakeholders		Ha: Perceptions of the environment change in the area of influence of TransMiCable.

*All measurements are conducted in intervention and control groups during baseline and 6–10 months after the inauguration of TransMiCable. MOVES app was used on the baseline, and Muévelo was used for follow-up. Semi-structured interviews for assessing the historical context were only conducted at baseline*.

Baseline measurements were collected in both intervention and control areas from February to November of 2018, before the inauguration of the TransMiCable in December 2018. The follow-up measurement is planned to be conducted from July to November 2019.

### Measurements

#### Sampling of Individuals and Environmental Units

The study population comprised adults aged 18 and older, without known cognitive disability, who have lived in the study area (intervention or control) for at least 2 years, who were not planning to move to another neighborhood that is not part of the study area within the next 2 years and who accepted to wear the accelerometer. Participants who opted to participate received an incentive of a tote bag and a 3 USD gift card, and participants who successfully completed the use of the accelerometer to measure physical activity participated in a lottery for a 150 USD gift card at the end of each measurement. The same incentives will be used during the follow-up period. The sample size of individuals was powered to detect changes equivalent to standardized mean differences (d) in outcomes that range from 0.3 to 0.4. We, therefore, aimed to achieve a sample size of 800 adults in each group with a response rate at follow-up of 70%, which will provide a power of ≥80%. To achieve the sample size, we selected a sample of 453 blocks within each buffer (225 from the intervention buffer, and 228 from the control buffer) with probability proportional to the density of the parcels in each block. Within each block, we selected households with a probability proportional to the number of parcels. Within each household, we enumerated adults and verified inclusion criteria. Among the adults who fulfilled the selection criteria, we randomly selected one adult using a random number table.

Additionally, we oversampled 200 adults, by selecting blocks randomly, in the intervention group, around the most distal stations, which are expected to be most impacted by the TransMiCable implementation. All individuals completed a questionnaire. All individuals were eligible for using accelerometers but, due to the limited amount of accelerometers, 85% of participants wore the accelerometers for baseline measurement in intervention and control groups. All individuals were asked to use MOVES, but only those who had smartphones and accepted to use the GPS information from the smartphone, and were interviewed before June 30 (the app was removed from Facebook servers on that date) used the app for baseline measurement in intervention and control groups. As a result, 15% of the participants used the app MOVES for baseline. At the follow-up, all eligible individuals will be asked to use our app “Muévelo” (in English “Move it”). “Muévelo” is an Android mobile app, created by our research team, to overcome some of the issues we experienced with MOVES, in particular, we wanted “Muévelo” to be able to deal with the limitations and specific conditions imposed by the social and technological conditions of the targeted users and their context. Therefore, “Muévelo” was design considering: (1) low battery consumption through a passive collection of GPS locations by using an adaptive sampling algorithm, (2) compatibility with a broad set of mobile devices and Android versions, and (3) offline-mode data collection and automatic opportunistic synchronization with a backend server when WI-FI connection is available. In addition, a random sample of 350 participants in the intervention area was selected for surveys to assess stated transportation preferences and satisfaction with the public transport modes in the area of TransMiCable.

#### Parks

We selected two parks (Illimani and Manitas) that will be intervenedas part of the TransMiCable improvement package in the area. Illimani is the most distal park in the studied area, and Manitas is a park that is projected to be equipped with an outdoor gym. The control parks in San Cristóbal, Parque Moralba, and Parque La Victoria, were selected to be similar to the intervention parks in area and type of park (i.e., neighborhood and zonal) (Figure 3B).

#### Transport Micro-Environments

We selected the three main transport modes in the control and intervention areas according to the Bogotá Household Travel Survey (44) and assessed the exposure to air pollution through visits by study staff designed to mimic the usual travel patterns of adults in the control and intervention areas. Specifically, air pollution was measured as follows: (1) at the roadside passenger waiting area of the stations of the BRT system, (2) on the vehicle of the main feeder line to the BRT system, (3) on a regular bus serving each area, and (4) a semi-formal public transport vehicle. The unit of analysis corresponds to each micro-environment of transport. Therefore, we conducted five measurements per transport micro-environment in the intervention (*N* = 20) and control areas (*N* = 20) (Figures 3A,B).

### Variables and Measurements

#### Environmental and Social Determinants of Health

##### Exposure to air pollution in micro-environments of transport

We assessed personal exposure to fine particulate matter (PM2.5), equivalent black carbon (eBC), and carbon monoxide (CO) by directly measuring the concentrations of those parameters using portable devices in four different transport micro-environments. The instruments used to measure PM2.5 concentration are a photometric particulate matter sensor (DustTrak models 8520 and 8530, TSI Inc. MN, USA), and two gravimetric PM2.5 samplers Personal Environmental Monitors (PEM) (SKC Inc. PA, USA). We determined personal exposure to equivalent black carbon (eBC) with portable Aethalometers (AE51, MicroAeth, CA, USA). To measure carbon monoxide concentrations, we used an electrochemical cell sensor (DeltaOhm, P37AB1347 SICRAM probe). All of these devices are carried in a backpack by individuals traveling in the four transport modes. The sampling inlets for these devices are located in the breathing zone of the individuals carrying them. The time resolution of the direct-reading instruments was set to 10 s. To account for day-to-day background variations in ambient air pollution levels, we used hourly data from the air-quality monitoring network of Bogotá for the specific dates and times of the micro-environmental samplings (20).

Physical activity levels of the individuals carrying the portable air pollution measuring devices were measured with accelerometers (Actigraph GT3X+, Ft. Walton). Measurements were conducted in each microenvironment of transport on weekdays during morning and afternoon commuter peak and valley hours (7:00 to 10:00 a.m. and 4:30 to 7:30 p.m.). The same measurements of air pollution in microenvironments, plus adding the measurement at the roadside passenger waiting area of the TransMiCable stations and in TransMiCable cabins, will be conducted 10 months after the implementation of TransMiCable during the same month of the year.

##### Physical environment

We assessed the physical environment through different sources. First, individuals completed a questionnaire that includes items related to the perceptions of the neighborhood. This questionnaire is a compilation of questions obtained from two sources, the Development Bank of Latin America-CAF survey (45) and the Encuesta Multipropósito (46). Second, we used secondary data from the Spatial Data Infrastructure for Bogotá (IDECA) to measure built environment attributes, including the proportion of area devoted to recreational and cultural facilities and the proportion of area for community centers and local markets in both study areas. Third, we applied the System for Observing Play and Recreation in Communities (SOPARC) and the Physical Activity Resource Assessment (PARA) instruments to assess PA levels and equipment quality of four parks (47). We conducted these measurements for seven days per park in the morning (9:00 a.m.-12 p.m.) and the afternoon (1:00–4:00 p.m.) during both weekdays and the weekend. We plan to collect the survey measurements of the perceptions of the neighborhoods 6 to 10 months after the implementation of TransMiCable according to fieldwork logistics. SOPARC and PARA measurements will be conducted 10 months after the implementation of TransMiCable to have comparable weather conditions with baseline measurements.

##### Transport accessibility

We assessed transport accessibility through four sources. First, we applied an individual questionnaire that includes items related to travel time, costs, trips, and modal choice. This questionnaire is a compilation of items obtained from the Bogotá Household Travel Survey administered by the local administration in 2011 (48) and 2015 (44), and the Development Bank of Latin America-CAF survey (45).

Second, in a convenience sample of 301 individuals in the intervention and control sites who owned a smartphone and accepted to participate in this protocol, we installed a mobile app on the participants' personal smartphones called MOVES by Facebook to passively record their travel patterns for at least seven consecutive days (plus an initial familiarization day and the morning of the final day). Data produced by this app included a timeline with information about places and movements that is geo-referenced. The MOVES app was suddenly withdrawn from the app stores in July of 2018. Therefore, we designed and implemented our own Android mobile app Muévelo that will be used in the follow–up period. Muévelo uses Google services for geo-location and detects the travel mode (walking, car, running). By design, Muévelo supports a wide set of mobile devices and could be used even when no mobile data or internet connection is available.

Third, in a subsample of 350 randomly selected participants who completed the survey in the intervention group, we collected stated preference data and satisfaction of participants with the public transport modes. We collected these data through a questionnaire collected 1 month before the implementation of TransMiCable. Fourth, we used fare card data from the bus rapid transit system (TransMilenio) from all number of boardings of TransMilenio to estimate and examine the evolution of the origin-destination matrix over time in Bogotá including the nearest stations of intervention and control areas. Finally, we plan to estimate changes in the Transit Opportunity Index (TOI) (49, 50) after the intervention to quantify the accessibility and connectivity of the users of TransMiCable. The TOI will serve as a proxy to measure transport equity (or lack thereof) by comparing access to the public transport system in the TransMiCable area against other areas of the city. We will collect all measurements six to 10 months after the implementation of TransMiCable, except for the stated preferences that were collected only for the baseline period.

##### Employment, leisure time, and social capital

We assessed these social determinants of health through the questionnaire. We compiled employment and leisure time indicators from Encuesta Multipropósito (46). We measured the construct of social capital (SC) by using an adapted questionnaire based on three different sources: the World Bank (SC-IQ) (51), the Development Bank of Latin America-CAF survey (45), and Encuesta Multipropósito (46). This set of questions assesses the domains of social networks, social norms, trust, and reciprocity. We will collect the same measurements 6 to 10 months after the implementation of TransMiCable.

#### Physical Activity

##### Leisure and transport physical activity

We assessed Leisure and transport physical activity through two sources. First, we collected an individual questionnaire—the International Physical Activity Questionnaire (IPAQ)-long form (52), which includes transport and leisure-time PA dimensions. IPAQ has been validated in Colombia through accelerometry (53). Second, most of the individuals who responded to the survey were asked to wear an ActiGraph GT3X or GT3X+ accelerometer at the waist and positioned in line with the right mid-axillary line for awake hours on at least seven consecutive days (plus an initial familiarization day and the morning of the final day). Accelerometers were initialized to collect data at 60-s epochs. The same measurements of physical activity will be conducted 6 to 10 months after the implementation of TransMiCable.

#### Health Outcomes

##### Health-related quality of life

We measured Quality of life (QOL) with the WHOQOL-brief instrument (54). This 26-item questionnaire has four separate scores, which correspond to the following domains: physical health, psychological health, social relationships, and the environment. The WHOQOL has been used previously in the Americas (54) and has been validated in the Colombian population (55).

##### Respiratory diseases

We assessed respiratory diseases of children <5 years old and adults more than 60 years old—two subgroups who are particularly vulnerable to exposure to air pollution from two sources: first, we applied a questionnaire that includes items about the presence of respiratory symptoms in children and older adults living in the selected households. Second, we calculated the incidence of respiratory diseases at a neighborhood level using the records of acute respiratory disease cases reported for children <5 years old and older adults. We retrieved the data for respiratory diseases from the Colombian National Public Health Surveillance System SIVIGILA (Sistema Nacional de Vigilancia en Salud Pública).

##### Crime

We will assess crime indicators (thefts, robbery, and homicides) by retrieving secondary data from the National Police Records. We will calculate the crime rates per habitant at the neighborhood level for the intervention and control areas.

We will collect the same measurements of health outcomes 6 to 10 months after the implementation of TransMiCable.

### Qualitative Component

We conducted a qualitative assessment component in both intervention and control groups to understand the historical context of the intervention, to assess the changes in environmental and social determinants of health, and to facilitate a consensus and advocacy-building process among community residents, policymakers, and researchers.

During the protocol design phase, we conducted semi-structured interviews to assess the community's perspectives regarding the potential health impacts of TransMiCable and to understand its historical context. Then, we employed the citizen science “by the people” Our Voice model (56), which entailed four stages, as follows: (1) planning and recruitment; (2) community walks using the Stanford Healthy Neighborhood Discovery Tool (57); (3) review of themes that emerged from the community-walks to prioritize issues and identify resources and potential partners; and (4) engaging residents in meetings with local public and private stakeholders to guide practical solutions. In stage 1, we recruited a subsample of 28 community leaders and community residents from the intervention and control areas. In stage 2, we used the Healthy Neighborhood Discovery Tool (DT), which is a user-friendly mobile app developed by the Stanford Healthy Aging Research and Technology Solutions Laboratory at Stanford University School of Medicine, USA (57). This app enables residents to document neighborhood features through geocoded photographs, audio narratives, and GPS-tracked walking routes. In stage 3, we conducted meetings with the citizen scientists, one for each intervention and control areas, with the aim of coding and synthesizing the data. We asked the participants to review the themes and categories that emerged from the walks and build consensus around the main problems and facilitators of the transport and residential environments for healthy living. The same stages will be conducted 14 months after the implementation of TransMiCable. Finally, in stage 4, we will have meetings with stakeholders in which the citizen scientists present their findings and, together with the stakeholders, brainstorm potential practical solutions.

### Planned Statistical Analysis

Our statistical analytical strategy involves several steps. First, we will test the pre-intervention balance between intervention and control groups on outcomes and covariates. Second, to measure change over time, we will fit hierarchical generalized linear models for each outcome on pre-and post-intervention data. Third, we will use a combination of difference-in-differences with propensity score matching techniques to contrast changes over time in intervention and control groups while tightly controlling for eventual imbalance in covariates to the extent possible (58).

We will conduct specific analyses to create the indicators of air pollution microenvironments, park renovation, transport accessibility, accelerometry to measure physical activity and secondary data on crime, and respiratory diseases.

#### Exposure to Air Pollution in Micro-Environments

First, we will calculate the mean concentration of PM 2.5, eBC, and CO in the four transport microenvironments before and after the intervention. We will apply a standard correction of the eBC concentration due to a decrease in instrument sensitivity as filter loading increases (59). We will synchronize the signals from all the measuring devices through a lagged cross-correlation analysis. Second, using the travel time data, modal share distribution data, and PA levels associated with each mode, we will calculate the inhaled dose of PM 2.5, eBC, and CO in current transport alternatives (60).

#### Built Environment and Parks

For the analysis of the built environment characteristics, we will calculate the proportion of areas covered with recreational and cultural facilities, community centers, and local markets. The systematic analysis of parks using SOPARC and PARA includes the following steps. First, we will describe the characteristics of the parks and target areas (quantity, size, and quality), and user characteristics (sex, age, and PA levels). Second, we will calculate the overall number of park visitors, the number of visitors being sedentary, and the number of visitors being moderately to vigorously active.

#### Transport Accessibility

The analysis of the travel patterns with the mobile app data includes the following steps. First, we will create a synthetic week. To consider the valid mobility data for a participant, data had to be available a minimum of 4 days (including a weekend day) with at least 10 h of recorded time per day. Second, and following similar analyses using GPS data to identify trips, transfer to other modes, and destinations (61). We will define a time threshold for each mode (BRT, regular buses, feeder, and lines). This time-threshold will be defined according to the average waiting time in bus stations reported in the 2015 Bogotá Household Travel Survey from only low-income households (44). The mode of transport will be determined based on the speed and distance to the nearest transit stop and transit stop locations for the city's BRT and regular buses. Third, we will identify places and trips based on the location and time spent. Fourth, we will calculate the average trip time and the average number of trips per day per participant. Fifth, we will calculate the average number of visits to recreational, health centers, and educational facilities by overlaying the city's official cartography with the places visited.

For the analysis of the secondary data from the fare cards of TransMilenio, we will estimate an OD matrix over time. We will calculate the TOI using the GTFS files of the system as input and our own Python tool that computes the TOI for large-scale public transport systems (with transfers).

For the analysis of the stated preference data and the current transport mode used, we will estimate discrete choice models to determine subjective values of the time and the propensity to use TransMiCable before its implementation.

#### Physical Activity

The analysis of the accelerometry data will include the following steps: We will create a synthetic week. For wear-time validation, a minimum of 3 week-day days and a minimum of 1 weekend day with at least 10 h of wear time will be required of each participant. Accelerometry data will be scored using Freedson cut-points for adults (62).

#### Qualitative Analysis

After the citizen scientists collect data using the Stanford Discovery Tool, the research team will prepare documents with verbatim transcriptions of each participant's audio narratives paired with their respective geo-tagged photographs. Participants will receive printed copies of their own data and will collectively review their photos and audio transcripts to identify relevant issues to address in their neighborhoods. After all community meetings are held, the research team will organize into clusters the similar categories that were produced in each of the community meetings. We will use a Chorematic representation (i.e., a pictographic representation of a territory) of the contexts of the neighborhoods in the most distal station of TransMiCable to summarize the findings (63). We will analyze the data from the community meetings with local policymakers by constructing narrative matrices by the theme that emerged during the collective discussions.

In addition, to increase the robustness of the results (64), we will follow a convergent mixed methods approach to triangulate qualitative and quantitative data sources. In our triangulation strategy, we will use the sections of the core questionnaire regarding perceptions and satisfaction with the neighborhood, along with the emergent themes of the qualitative analysis conducted by the subset of residents who participated in the Stanford Discovery Tool qualitative data collection. We will contrast quantitative and qualitative data for each relevant neighborhood perception outcome (e.g., safety, aesthetics) only use of saturated emergent themes will be included in the triangulation.

## Discussion

TRUsT is an international multidisciplinary study that is part of the SALURBAL study. To our knowledge, this is the first time a study will assess the effects of a cable car system on important environmental and social determinants of health and will use participatory citizen science methods to identify, prioritize, and communicate the most salient negative and positive aspects of the impact of this transport innovation. The study also uses an integrated knowledge translation approach developed and implemented in close consultation with intersectoral stakeholders and community members.

The study also presents several important challenges. The first challenge is the implementation of a timely and robust quantitative and qualitative evaluation of an intervention that is outside the control of the researchers and whose timing and components can vary based on the political context. To address this challenge, we collected baseline data in a timely manner in both intervention and control sites before the intervention and the team will be carefully monitoring intervention components. TransMicable started operating in December 2018, and currently, the park renovations and administrative services are being implemented, but there is uncertainty on the implementation of one of the two parks where we planned to collect data. Constant communication with TransMilenio SA regarding the implementation of the TransMicable project has helped in prioritizing components of the evaluation.

The second challenge will be attrition in our longitudinal study, partly the result of neighborhood gentrification and resident displacement caused by the rent and property value increases due to the intervention itself. To overcome this challenge we have followed these strategies: (1) we included only adults who do not plan to move to another neighborhood within the next 2 years; (2) we are giving the participants incentives; (3) we increased trust in the community by providing reports of people's physical activity levels and nutritional status and had meetings with community leaders; (4) we collected contact data from at least two relatives or friends participants and from social media (Facebook, Instagram, Twitter) to allow follow-up with participants; (5) we will follow up all the participants if they live in Bogotá, but for logistical and cost reasons we are not able to follow individuals who move away from the city; and (6) we included a module in the survey and in the qualitative component to assess the reasons for out-migration We will explore analytical alternatives to characterizing any bias associated with differential attrition.

The third challenge is related to objective measurements. We have limited numbers of accelerometers; therefore, we were only able to obtain accelerometer data on 85% of respondents' limiting power. Furthermore, the sudden end to the MOVES app means that we used two different data sources for measuring mobility behaviors and transport trajectories. The team prioritized the data collection with MOVES to complete data collection for the baseline and then developed “Muévelo” for the follow-up measurement. Although using different apps will pose challenges comparing trajectories, the self-reported questionnaire will help to identify key differences.

The fourth challenge is related to personal security issues for study staff in the area of evaluation. To overcome this challenge, we conducted multiple meetings with community leaders to develop trust and exchange of the progress of the evaluation. Community members reviewed the geographic location of the sample and provided safety recommendations for specific neighborhoods. When a neighborhood was considered highly unsafe for the research team, community members provided guidance and support to interviewers. Also, community leaders have been invited to seminars at the Universidad de Los Andes to provide their ongoing insights and perceptions regarding the TrUST study.

The last challenge pertains to integrating various disciplinary perspectives. To overcome this challenge, the research team developed the conceptual framework and the research protocol together. Furthermore, researchers from diverse disciplines have regular meetings together for analyses and interpretation of preliminary results. These results, in turn, are presented regularly to researchers from different disciplines as well as to the community and policy stakeholders.

The limitations noted above are inherent in the evaluation of any natural experiment. Despite these limitations, evaluations like TrUST involving a longitudinal controlled design, combining quantitative and qualitative approaches, and engaging community members and policymakers throughout, are critical to the development of urban policies that promote health and environmental sustainability in similar cities. The results of this study will provide valuable information and insights to policymakers in order to identify, formulate, and address strategies to ameliorate inequalities in highly segregated populations through sustainable transport. TrUST could also serve as an example for assessing the benefits as well as the unintended consequences of cable cars in other urban contexts.

## Ethics Statement

The studies involving human participants were reviewed and approved by Ethics Committee at The Universidad de Los Andes (Acta No.806 – 2017) and its amendments (Acta No. 977 – 2019, Acta No. 994 – 2019). The patients/participants provided their written informed consent to participate in this study.

## Author Contributions

All authors contributed to the design of the study and contributed to drafts of the manuscript. OS, DH-M, MW-M, and AD drafted the manuscript and coordinated the development of the project. LG, DR, JA, CM, and AM designed the transport protocol. RM and DM designed the air pollution protocol. CB, ML-V, and MA designed the MOVES protocol and the development of the Muévelo app. EM-H contributed to the designed of the protocol for QOL, and social capital. FM, JM, and AU designed and analyzed the GMB workshop. EG contributed to the design of respiratory health. CT, MW-M, DH-M, OS, and AK designed the protocol of Citizen's Science. PH, AD, DH-M, and OS contributed to the design of the sample. All authors have read and approved the drafts of the final manuscript.

### Conflict of Interest

The authors declare that the research was conducted in the absence of any commercial or financial relationships that could be construed as a potential conflict of interest.

## Abbreviations

BRT: Bus Rapid Transit
TrUST: Transformaciones Urbanas y Salud, El caso de TransMiCable en Bogotá Colombia
SALURBAL: Salud Urbana en America Latina
PM2.5: Particulate Matter 2.5 μm
eBC: Equivalent Black Carbon
CO: Carbon Monoxide
PEM: Personal Environmental Monitors
MVPA: Moderate to Vigorous Physical Activity
SOPARC: System for Observing Play And Recreation in Communities
PARA: Physical Activity Resource Assessment
OD: Origin – Destination
TOI: Transit Opportunity Index
SC: Social Capital
IPAQ: International Physical Activity Questionnaire
QOL: Quality Of Life
SIVIGILA: Sistema Nacional de Vigilancia en Salud Pública
DT: Discovery Tool
HGLMs: Hierarchical Generalized Linear Models
DID: Difference-in-Differences
PSM: Propensity Score Matching
SITP: Sistema Integrado de Transporte Público.
