# Supplementary material for: Urban Transformations and Health: Methods for TrUST—a Natural Experiment Evaluating the Impacts of a Mass Transit Cable Car in Bogotá, Colombia
Source: Front Public Health. 2020 Mar 10;8:64. doi: 10.3389/fpubh.2020.00064 (PMC7075807; doi:10.3389/fpubh.2020.00064)
Supplement: Supplementary file 1 [file Data_Sheet_1.docx]

**Appendix 1: Feedback loops from the causal loop diagram depicting the conceptual framework of TrUST**

|  | Feedback loops | Description |  |
| --- | --- | --- | --- |
| B1 |  | An increase in socio-economic conditions due to informal housing availability. The increase in informal housing availability leads to an increase in population density. Having more people in the area increases socioeconomic development, which favors the quality of life that in turn triggers informal housing availability in the area |  |
| R2 |  | An increase in the local infrastructure due to the new informal housing availability. The increase in informal housing availability promotes the development of greater local infrastructure, which improves the quality of life and promotes the generation of new informal housing. |  |
| B3 |  | A decrease in the local infrastructure due to the increase in population density. The supply of informal housing increases the population density of the area, which reduces the level of service of the local infrastructure. To the extent that local infrastructure worsens people's quality of life and the supply of informal housing. |  |
| B4 |  | A decrease in the quality of life due to the environmental impact of activities in the area. The increase in informal housing promotes pollution of rivers and streams in the area, which increases the environmental impact. Given this increase, people see their quality of life worse, which reduces the informal housing availability. |  |
| B5 |  | A decrease in the quality of life due to inequality in access to services. Informal housing increases local infrastructure, which creates a saturation of public services, increasing inequality of access. Given an increase in inequality, the quality of life is worsened, reducing informal housing. |  |
| B6 |  | A decrease in air quality due to the use of alternative transport systems. Congestion in the area promotes the use of alternative transport systems that generate more pollutants and worsen air quality. Poor air quality affects health conditions that worsen people's quality of life. Poor quality of life in the areas reduces informal housing and population density in the area, which reduces transportation congestion. |  |
| R7 |  | An increase in waiting time due to transport congestion. An increase in transport congestion causes people's waiting time to be increased. As the waiting time increase, people remain to wait longer for transportation, which increases congestion again. |  |
| R8 |  | An increase in waiting times due to the poor quality of the TransMiCable. The increase in congestion causes the quality of the TransMiCable to be hindered, which increases people's waiting time. As for waiting time increases, congestion increases. |  |
| B9 |  | An improvement in the quality of life due to low displacement and segregation in the area. As the quality of life worsens, informal housing decreases, which reduces population density. A reduction in population density leads to a reduction in the socio-economic development of the area. Less socio-economic development reduces the population diversity and tourism in the area. As tourism decreases, living costs decrease and reduce the displacement and segregation of people in the area, which improves the quality of life of people. |  |
| B10 |  | A decrease in population diversity in the system due to interurban displacement. The presence of greater population diversity in the area encourages greater interurban displacement, which has an impact on decreasing the socio-economic development of the area. As socio-economic development in the area is reduced, gender violence increases, and the quality of TransMiCable decreases. Given the low levels of quality, community appropriation is reduced as well as the safety in TransMiCable. When the TransMiCable is perceived insecure, the population diversity in the area is reduced. |  |
| R11 |  | An increase in socioeconomic development due to a positive environmental perception. When the territory perception is good, tourism in the area is increased. Increasing tourism increases the socio-economic development of the area and increases population diversity. A greater population diversity allows building a better territory perception. |  |
| R12 |  | A decrease in TransMiCable safety due to vandalism in the system. When the vandalism in the system increases, the security of the system is reduced. In the same way, by decreasing the security of the system, vandalism is increased. |  |
| R13 |  | An increase in gender-based violence due to low TransMiCable safety. Increasing the insecurity of the TransMiCable increases gender-based violence, which reduces the quality of the TransMiCable. When the quality of the TransMiCable is reduced, the community appropriation is reduced, which reduces the safety of the TransMiCable. |  |
| R14 |  | A decrease in gender-based violence for the wellness of people. An increase in people's wellness reduces gender-based violence in the system. This reduction increases the quality of the TransMiCable and thus improves social inclusion. By improving social inclusion in the system, people's wellness is increased. |  |
| R15 |  | A decrease in the quality of the TransMiCable by vandalism in the system. An increase in the vandalism of the system reduces security in the system, which makes it less accessible. When the accessibility of the TransMiCable is reduced, the quality too. Given the decrease in the quality of the TransMiCable, the community appropriation is also reduced, making vandalism to increase. |  |
| R16 |  | An increase in the community appropriation for the wellness of people. When social inclusion in the system increases, people see their wellness increased, which increases the community appropriation. Given an increase in the community appropriation, the accessibility of the TransMiCable increases and improves the quality of the TransMiCable. When the quality of TransMiCable is increased, social inclusion and social capital in the system increases. |  |
| B17 |  | Stronger gender violence generated by the demand for TransMiCable due to service quality. |  |
| R18 |  | An increase of security in TransMiCable by the community appropriation. Increasing security in the system improves accessibility and quality, thus, promotes more community appropriation. |  |
| R19 |  | An increase of TransMiCable quality by improving accessibility. TransMiCable quality promotes appropriation for the system, which improves accessibility. |  |
| R20 |  | An increase of community appropriation by access to TransMiCable development. |  |
| R21 |  | An increase of community appropriation by community participation. |  |
| R22 |  | An increase of cultural spaces in TransMiCable infrastructure because of the system quality. The increment of public and cultural spaces promotes active involvement and appropriation by the community. This appropriation favors systems accessibility, which improves TransMiCable quality. |  |
| B23 |  | A decrease in TransMiCable quality by congestion. Improvement in quality promotes a higher user demand, which congests the system and deteriorates the quality. |  |
| B24 |  | A decrease in health conditions due to road accidents. The worsening of health conditions promotes a greater demand for TransMiCable infrastructure and urban projects and programs. These strategies increase the quality of TransMiCable and reduce road accidents. |  |
| R25 |  | Increase in mental health by incorporating healthy habits. |  |
| B26 |  | A decrease in health conditions due to mental health. By worsening people's health conditions, worse healthy habits that affect mental health are promoted. |  |
| R27 |  | Improvement of health conditions by the incorporation of healthy habits. |  |
| R28 |  | Increase in health conditions due to the increase in physical activity. |  |
| R29 |  | Increase in physical activity by creating more spaces for recreation and sports. To the extent that the quality of the TransMiCable improves, there is the opportunity to create new spaces for recreation and sports that encourage the improvement of physical activity. To the extent that people are more physically active, they require TransMiCable of a greater amount of infrastructure related to spaces for recreation and sports, and these demands can eventually lead to the development of new urban programs and projects by TransMiCable generating an improvement in the quality of TransMiCable. |  |
| R30 |  | A decrease in health conditions due to alcohol and drug use. As health conditions decrease, the demand for infrastructure in TransMiCable increases leading to the development of more urban projects and programs in TransMiCable. This increase leads to an increase in the quality of the TransMiCable, which decreases the waiting time, which increases the rest time and promotes the consumption of alcohol and drugs. |  |
| B31 |  | A decrease in the quality of life due to family time. The decrease in people's quality of life leads to a decrease in informal housing availability, which decreases the population density in the area. This decrease leads to less congestion in the area, and people spend less time waiting for transportation. As the waiting time decreases, the leisure time increases, and the family time increases. |  |
|  | Feedback loops starting with R are Reinforcement loops  Feedback loops starting with B are Balance loops | | |
